# Supplementary material for: On the effect of inheritance of microbes in commensal microbiomes
Source: BMC Ecol Evol. 2022 Jun 16;22:75. doi: 10.1186/s12862-022-02029-2 (PMC9204957; doi:10.1186/s12862-022-02029-2)
Supplement: Supplementary file 1 — Additional file 1: Fig. S1. Occurrence of a microbial taxon in hosts under microbial inheritance. Fig. S2. Microbial load distribution across a host population, with or without microbial inheritance. Fig. S3. Frequency of a microbial taxon distribution across the host population, with or without inheritance. Fig. S4. Average frequency of a microbial taxon in hosts under microbial inheritance. Fig. S5. Effect of asymmetric inheritance on hosts with longer lifespans. Fig. S6. Difference in microbial load between ‘low’ and ‘seed-like’ inheritance. Fig. S7. Difference in the frequency of a microbial taxon between ‘low’ and ‘seed-like’ inheritance. [file 12862_2022_2029_MOESM1_ESM.pdf]

Supplementary Material: On the effect of inheritance of microbes in commensal microbiomes

Román Zapién-Campos[1], Florence Bansept[1], Michael Sieber[1], and Arne Traulsen[1,*]

[1]Max Planck Institute for Evolutionary Biology, Plön, Germany

[1]zapien@evolbio.mpg.de, bansept@evolbio.mpg.de, sieber@evolbio.mpg.de

[*]Corresponding author: traulsen@evolbio.mpg.de

A Supplementary methods

A.1 Deterministic and stochastic components of the model

We have introduced a model of the microbiome dynamics where we track the frequencies of a taxon i, x_i, and the set of other taxa, o_i; together, the vector $\mathbf{x} = \{x_i, o_i\}$. In Eq. (5) we expressed the model in the form of a stochastic differential equation – that describes the microbial dynamics within a host during its lifespan– where the deterministic, $\mathbf{A}[\mathbf{x}]$, and stochastic, $B[\mathbf{x}]$, contributions were introduced. Changes have magnitude $\frac{1}{N}$. The deterministic part is given by the expected change of $\mathbf{x}$ that results from the transition probabilities in Eq. (1),

$$\mathbf{A}[\mathbf{x}] = \frac{1}{N}\frac{1}{1-\tau}\begin{bmatrix} T^{o_i-}_{x_i+} + T^{o_i}_{x_i+} - T^{o_i+}_{x_i-} - T^{o_i}_{x_i-} \\ T^{o_i+}_{x_i-} + T^{o_i+}_{x_i} - T^{o_i-}_{x_i+} - T^{o_i-}_{x_i} \end{bmatrix}. \tag{S1}$$

The stochastic part is related to the matrix of covariant change of $\mathbf{x}$:

$$V[\mathbf{x}] = \frac{1}{N^2}\frac{1}{1-\tau}\begin{bmatrix} T^{o_i-}_{x_i+} + T^{o_i}_{x_i+} + T^{o_i+}_{x_i-} + T^{o_i}_{x_i-} & -(T^{o_i+}_{x_i-} + T^{o_i-}_{x_i+}) \\ -(T^{o_i+}_{x_i-} + T^{o_i-}_{x_i+}) & T^{o_i+}_{x_i-} + T^{o_i+}_{x_i} + T^{o_i-}_{x_i+} + T^{o_i-}_{x_i} \end{bmatrix}. \tag{S2}$$

$B[\mathbf{x}]$ is the matrix that satisfies $B[\mathbf{x}]^T B[\mathbf{x}] = V[\mathbf{x}]$. This is calculated analytically [1] after defining

the quantities $w = \sqrt{\text{det}(V[\mathbf{x}])}$ and $d = \sqrt{\sum_i V[i,i] + 2w}$,

$$B[\mathbf{x}] = \frac{V[\mathbf{x}] + wI}{d}, \tag{S3}$$

where I is the identity matrix.

Note that Eq. (S1) and Eq. (S2) refer to the lifetime of a single host, therefore we divide by $1-\tau$ to remove it from each transition probability. We had introduced $1-\tau$ in Eq. (1) to explain the effect of host death at the population level.

A.2 Condition for deterministic increase of lineage taxa

We start from the definition of $\mathbf{A}[1]$, Eq. (S1). This equation indicates the deterministic change of frequency of a lineage taxon (x_i) as a function of x_i, other microbes frequency (o_i), and parameters of migration (m), frequency in the pool of colonizers (p_i), and how rapidly available space is occupied (α_0). Asking under which condition $\mathbf{A}[1] > 0$, leads to

$$T_{x_i+}^{o_i-} + T_{x_i+}^{o_i} > T_{x_i-}^{o_i+} + T_{x_i-}^{o_i}$$

Using the definition of the transition probabilities in Eq. (1) and simplifying, we find

$$(1-x_i)\left((1-m)\frac{x_i}{\alpha_0 x_0 + x_i + o_i}\right) > x_i\left(m + (1-m)\left(1 - \frac{x_i}{\alpha_0 x_0 + x_i + o_i}\right)\right)$$

where we used the fact that lineage taxa are absent from the pool of colonizers, $p_i = 0$. Simplifying and solving for $x_i + o_i = 1 - x_0$, we find

$$x_i + o_i < 1 - \frac{m}{1-\alpha_0} \tag{S4}$$

Thus, the growth of lineage taxa stops before the microbial load, $x_i + o_i$, reaches frequency 1, as this is constrained by migration, m, and how rapidly available space is occupied, α_0.

References

[1] Edward Allen. *Modeling with Itô Stochastic Differential Equations*. Springer, 2007.

B Supplementary figures

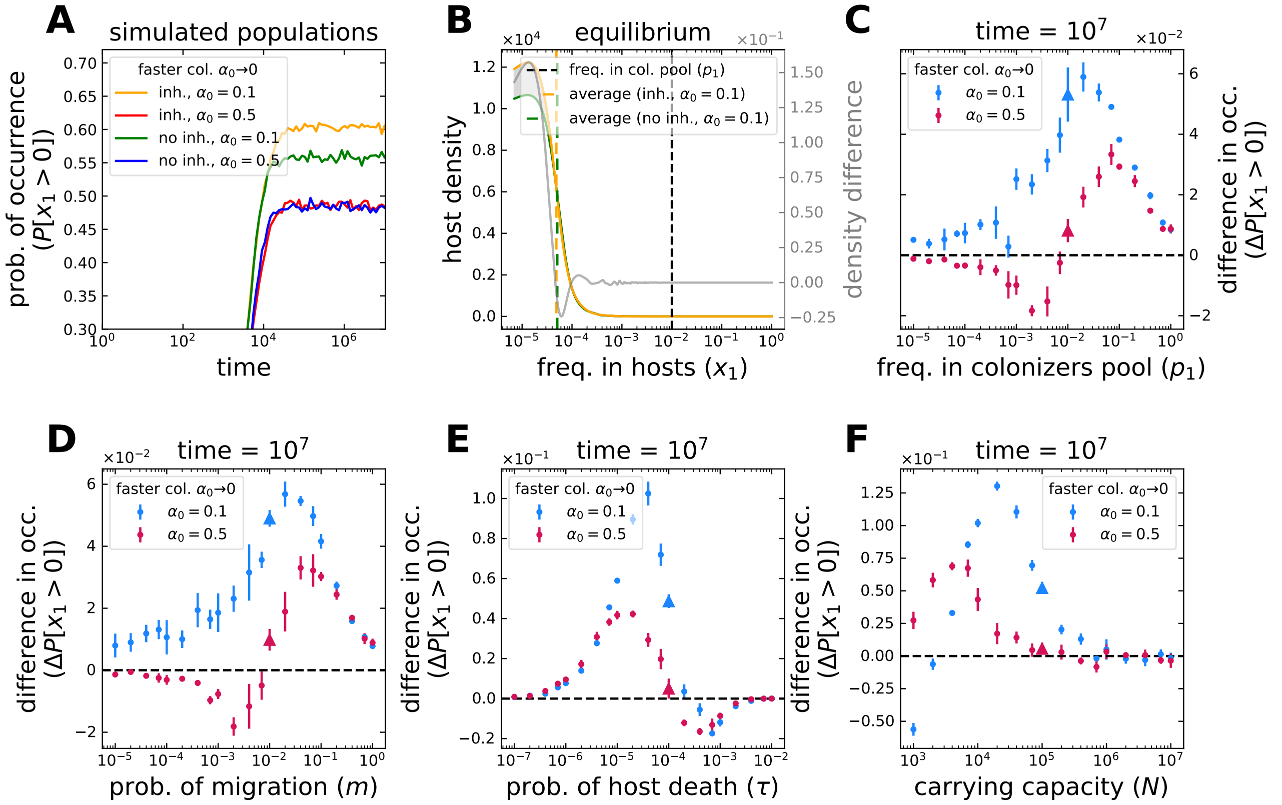

Fig. S1: **Occurrence of a microbial taxon in hosts under microbial inheritance.** We repeat the analysis from Fig. 2, but instead of load, $x_i + o_i$, we look into a single microbial taxon, x_i. (**A**) Starting from a condition where all hosts are initially empty, the microbial occurrence increases through time. In this particular case, inheritance increases the occurrence if hosts are colonized rapidly, $\alpha_0 \to 0$. (**B**) The hosts now contain the taxon in small frequencies. The cases shown in (A-B), with parameters $p_1 = 10^{-2}$, $m = 10^{-2}$, $\tau = 10^{-4}$, and $N = 10^5$, are indicated by the triangles in (C-F). (**C**) Changes are small for other frequencies in the pool of colonizers, p_1, but those at intermediate values benefit the most from inheritance. (**D**) The maximum change occurs for intermediate migration from the pool of colonizers, m. For $m \to 1$ the taxon colonizes hosts even without inheritance. Instead for $m \to 0$ the taxon does not colonize the hosts. (**E**) Larger changes occur for intermediate host death probabilities, τ, and fast colonization. Long living hosts, $\tau \to 0$, contain the taxon even without inheritance. Short living hosts, $\tau \to 1$, are less likely to be colonized by the taxon within their lifetime. (**F**) In contrast to the microbial load (Fig. 2E), for a single taxon the maximum change occurs at intermediate capacities for microbes, N. The change can be negative once inheritance favours more abundant taxa competing for limited space (see C-F). Points and bars in (C-F) indicate the average and standard deviation of 6 simulation pairs, with vs. without inheritance, with 10^4 hosts each. Offspring receive 9% of their parent's microbiome on average, $a_i = 0$ and $b_i = 9$ in Eq. (4). The whole distributions are shown in Fig. Sup. S3.

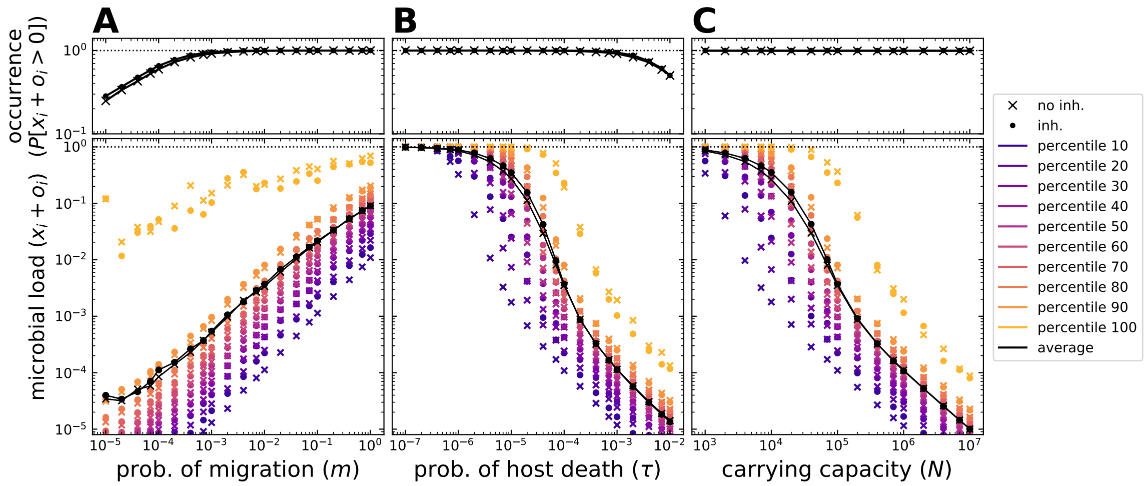

Fig. S2: **Microbial load distribution across a host population, with or without microbial inheritance.** The microbial load is the set of all microbes. In contrast to the difference between distributions, Figs. 2 and 3, here the distributions are shown. The cases without and with inheritance are indicated by $\times$ and •, respectively. Single parameters are modified from the condition $m = 10^{-2}$, $\tau = 10^{-4}$, and $N = 10^5$. The probability of occurrence and frequencies within hosts increase for (**A**) larger migration from the pool of colonizers, $m \to 1$, and (**B**) longer host lifespan, $\tau \to 0$. (**C**) While occurrence is constant at 1, frequencies increase for smaller capacities for microbes, N. Inheritance might increase both observables for certain parameter combinations and percentiles of the distribution (compare • to $\times$). The increase is evident for small percentiles. Decrease might occur for large percentiles. Only for $\tau \lesssim 2 \cdot 10^{-7}$ all hosts reach carrying capacity within their lifetime. Each simulation included 10^4 hosts and parameters $a_i = 0$ and $b_i = 9$ for inheritance, Eq. (4) – offspring receive 9% of their parent's microbiome on average – and $\alpha_0 = 0.1$ for available space occupation.

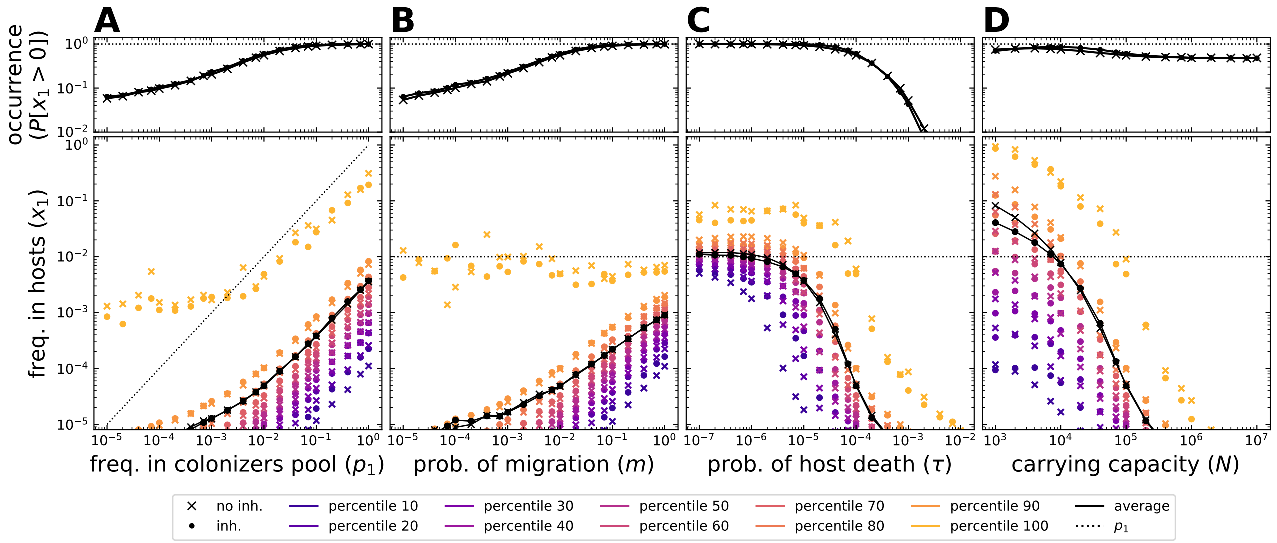

Fig. S3: **Frequency of a microbial taxon distribution across the host population, with or without inheritance.** In contrast to the difference between distributions, Figs. Sup. S1 and S4, here the distributions are shown. The cases without and with inheritance are indicated by $\times$ and $\bullet$, respectively. Single parameters are modified from the condition $p_1 = 10^{-2}$, $m = 10^{-2}$, $\tau = 10^{-4}$, and $N = 10^5$. (**A**) The probability of occurrence and frequency within hosts increase for higher abundances in the pool of colonizers, $p_1 \to 1$, and (**B**) larger migration from the environment, $m \to 1$. For $p_1 \to 0$, hosts with larger frequencies than in the pool of colonizers ($x_1 > p_1$) might occur stochastically. In contrast to microbial load (Fig. Sup. S2), inheritance might decrease the frequencies for (**C**) long host lifespans, $\tau \to 0$, and, (**D**) smaller capacities for microbes, N, where hosts are fully colonized. The reduced variability of the early microbiome, makes hosts with initially large frequencies of the microbial taxon less likely. Even if low frequencies increase, the average frequency decreases as a result. Inheritance increases the average frequency for intermediate values of τ and N, where hosts are partially colonized (Fig. Sup. S2 B-C). Each simulation included 10^4 hosts and parameters $a_i = 0$ and $b_i = 9$ for inheritance, Eq. (4) – offspring receive 9% of their parent's microbiome on average – and $\alpha_0 = 0.1$ for the available space occupation.

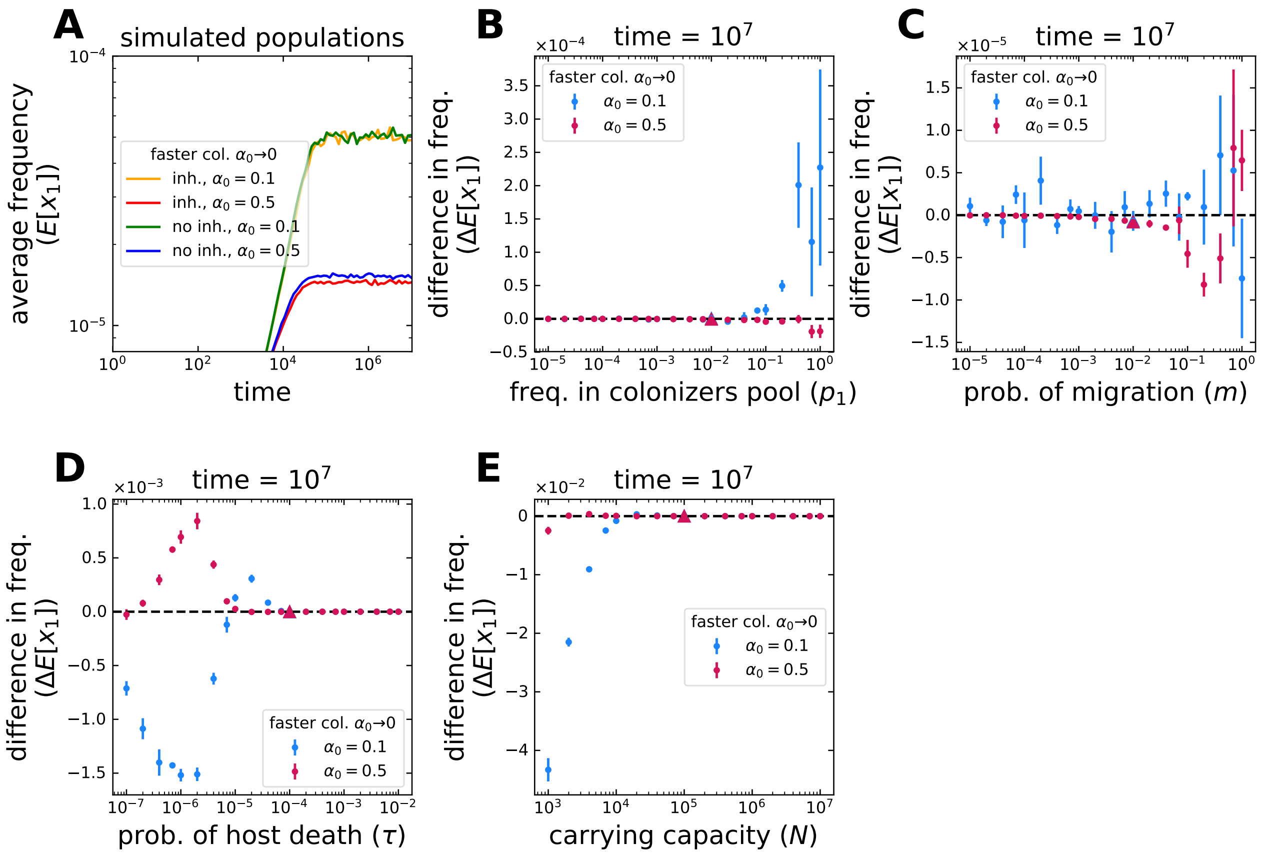

Fig. S4: **Average frequency of a microbial taxon in hosts under microbial inheritance.** We repeat the analysis from Fig. 3, but instead of load, $x_i + o_i$, we look into a single microbial taxon, x_i. (**A**) Starting from a condition where all hosts are initially empty, the average frequency of microbes in hosts increases through time before reaching an equilibrium. In this particular case, inheritance makes the average slightly larger if hosts are occupied more slowly, $\alpha_0 = 0.5$. Although more hosts harbour the taxon, no change occurs for $\alpha_0 = 0.1$, as inheritance reduces the variability between individuals. The cases shown in (A), with parameters $p_1 = 10^{-2}$, $m = 10^{-2}$, $\tau = 10^{-4}$, and $N = 10^5$, are indicated by the triangles in (B-E). (**B**) No changes occur for multiple frequencies in the pool of colonizers, p_1, and (**C**) migrations from the pool of colonizers, m. (**D**) The largest changes occur for intermediate host death probabilities, τ. For long living hosts, $\tau \to 0$, the change produced by inheritance can be negative; (**E**) similarly for small capacities for microbes, N, where inheritance causes abundant taxa to outcompete others. Points and bars in (B-E) indicate the average and standard deviation of 6 simulation pairs, with vs. without inheritance, with 10^4 hosts each. Offspring receive 9% of their parent's microbiome on average, $a_i = 0$ and $b_i = 9$ in Eq. (4). The whole distributions are shown in Fig. Sup. S3.

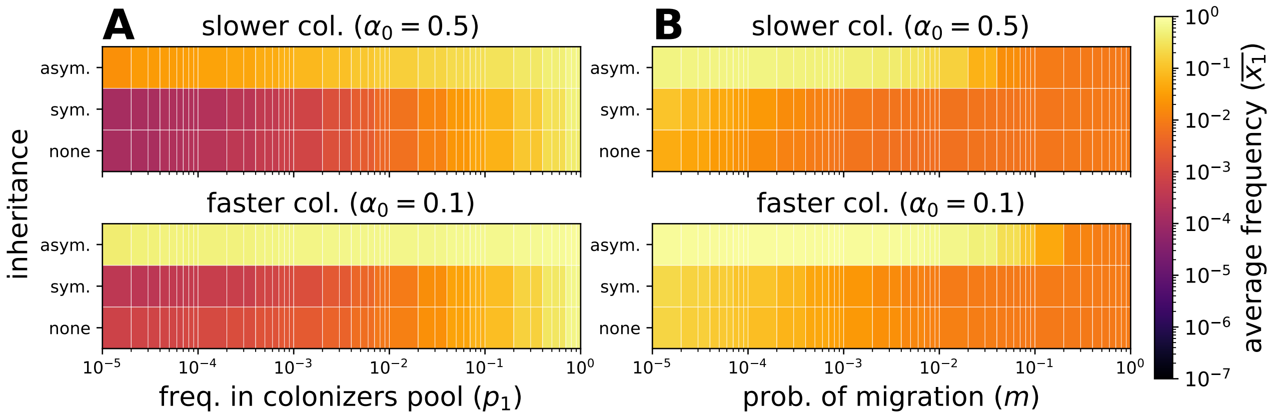

Fig. S5: **Effect of asymmetric inheritance on hosts with longer lifespans.** Cases without inheritance and inheritance are compared. Inheritance is symmetric if offspring receive 9% of their parent's microbiome on average ($a_i = 0$ and $b_i = 9$). Inheritance is asymmetric if offspring receive 9% of taxon 1 and 1% of other taxa ($a_i = 0$ and $b_1 = 9$, $b_{i \neq 1} = 99$ in Eq. (4)). Available space within hosts is occupied more easily for $\alpha_0 \to 0$. Single parameters are modified from the condition $p_1 = 10^{-2}$, $m = 10^{-2}$, $\tau = 10^{-6}$, and $N = 10^5$. As opposed to Fig. 4, where $\tau = 10^{-4}$, here hosts have longer lifespan ($\tau = 10^{-6}$). (**A**) The average frequency of a taxon increases for larger abundances in the pool of colonizers (p_1), especially if available space is occupied more rapidly. (**B**) A smaller immigration probability (m), preserves the effect of inheritance, leading to larger average frequencies. Asymmetric inheritance is highly influential in both conditions. Each simulation included 10^4 hosts.

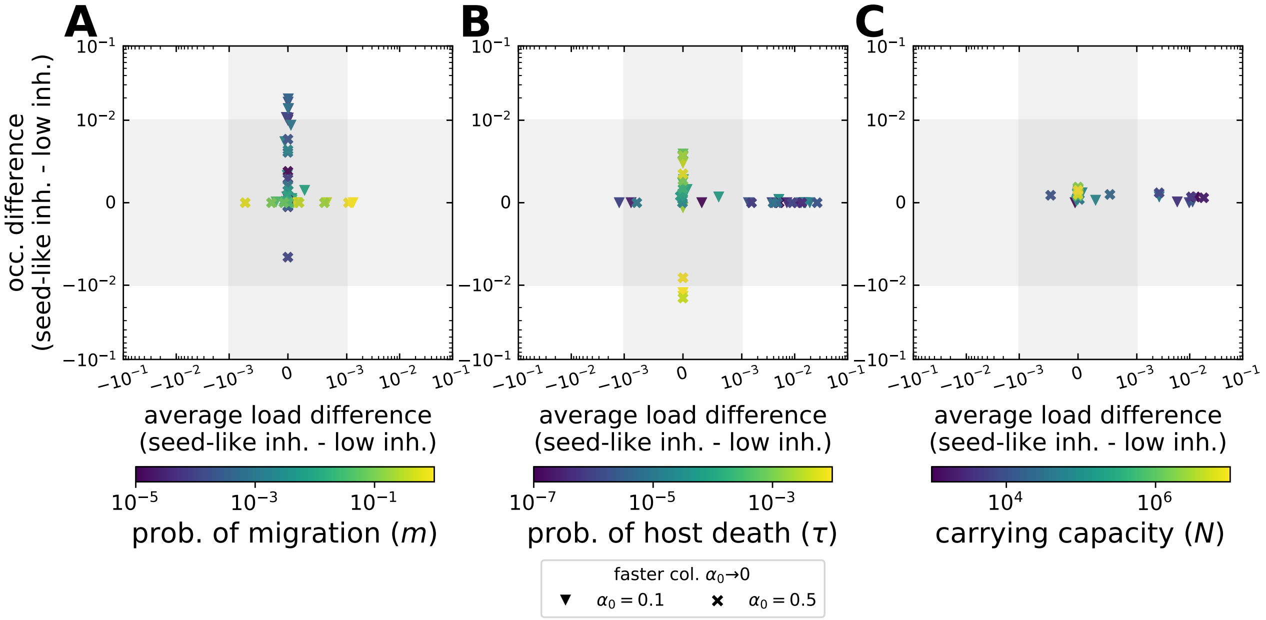

Fig. S6: **Difference in microbial load between 'low' and 'seed-like' inheritance.** A positive difference indicates the observable is larger for seed-like inheritance (Fig. 1B). For both, low and seed-like inheritance, offspring receive 9% of their parent's microbiome on average ($a_i = 0$ and $b_i = 9$ for low inheritance, and $a_i = 9$ and $b_i = 99$ for seed-like inheritance in Eq. (4)). Low inheritance corresponds to data shown in Fig. 2 and Fig. 3. Single parameters are modified from the condition $m = 10^{-2}$, $\tau = 10^{-4}$, and $N = 10^5$. (**A**) For low migration from the pool colonizers, $m \to 0$, seed-like inheritance increases the microbial occurrence (a exception stems from a slower occupation of available space, $\alpha_0 = 0.5$). For $m \to 1$, it mildly increases the average microbial load. (**B**) For low host death, $\tau \to 0$, this inheritance mode increases the average load importantly. For $\tau \to 1$, it only affects the occurrence, even decreasing it. (**C**) For varying carrying capacity (N), larger average loads are obtained for small N. Each point corresponds to the difference of observables calculated from simulations with 10^4 hosts. The scale of axes is logarithmic, but linear within $\left[-10^{-3}, 10^{-3}\right]$ for the average load, and $\left[-10^{-2}, 10^{-2}\right]$ for the occurrence.

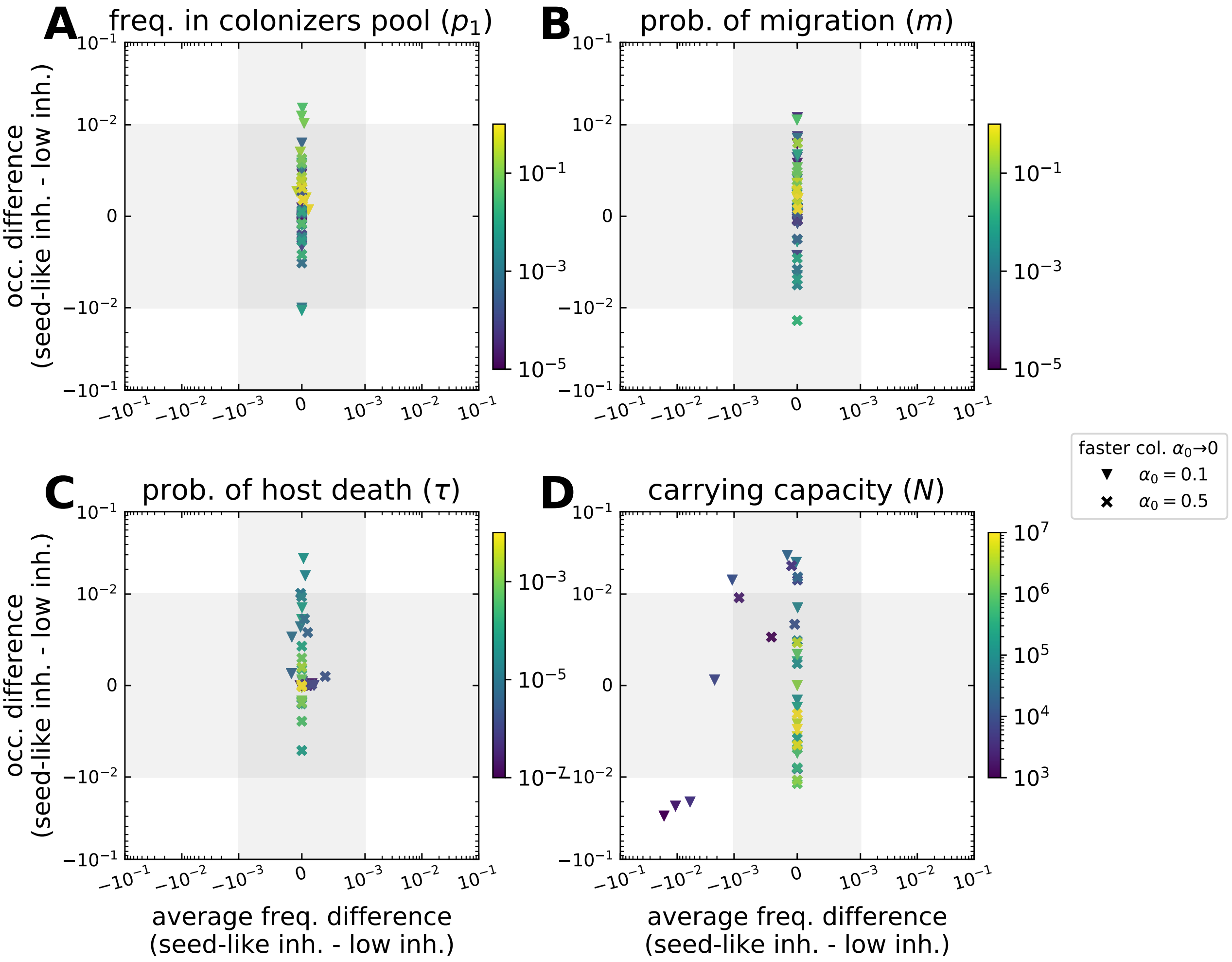

Fig. S7: **Difference in the frequency of a microbial taxon between 'low' and 'seed-like' inheritance.** A positive difference indicates the observable is larger for seed-like inheritance (Fig. 1B). For both, low and seed-like inheritance, offspring receive 9% of their parent's microbiome on average ($a_i = 0$ and $b_i = 9$ for low inheritance, and $a_i = 9$ and $b_i = 99$ for seed-like inheritance in Eq. (4)). Low inheritance corresponds to data shown in Fig. Sup. S1 and Fig. Sup. S4. Single parameters are modified from the condition $p_1 = 10^{-2}$, $m = 10^{-2}$, $\tau = 10^{-4}$, and $N = 10^5$. (**A**-**C**) A seed-like inheritance primarily modifies the occurrence for various values of frequency in the pool of colonizers (p_i), migration (m), and host death (τ). (**D**) For varying values of the carrying capacity for microbes (N), the main change is on the occurrence, however, for small N a decrease of average frequency is observed. A decrease or increase of occurrence is not clearly attributable to the rate of host colonization (α_0). Each point corresponds to the difference of simulations with 10^4 hosts. The scale of axes is logarithmic, but linear within $\left[-10^{-3}, 10^{-3}\right]$ for the average frequency, and $\left[-10^{-2}, 10^{-2}\right]$ for the occurrence.
